# Case Report: Vascular abdominal pain in systemic sclerosis—chronic mesenteric ischemia from high-grade superior mesenteric artery stenosis with coexisting aortomesenteric narrowing

**DOI:** 10.3389/fmed.2026.1882228

**Published:** 2026-07-24

**Authors:** Ji Li, Jing Zhang, Sheng-Guang Li, Lina Zhang, Yadan Zou, Ting Long, Ruohan Yu, Yanfeng Zhang

**Affiliations:** Department of Rheumatology and Immunology, Peking University International Hospital, Beijing, China

**Keywords:** abdominal pain, case report, chronic mesenteric ischemia, endovascular treatment, superior mesenteric artery stenosis, superior mesenteric artery syndrome, systemic sclerosis

## Abstract

Gastrointestinal involvement is common in systemic sclerosis (SSc), but severe abdominal pain should not be attributed automatically to dysmotility or malabsorption. We report a 72-year-old woman with long-standing limited cutaneous SSc who developed recurrent severe abdominal pain, progressive oral-intake limitation, and marked weight loss. Abdominal computed tomography angiography showed high-grade calcified stenosis at the superior mesenteric artery (SMA) origin together with a reduced aortomesenteric angle of 17 degrees and a distance of 4.7 mm, creating a coexisting SMAS-like anatomic context. After multidisciplinary review, endovascular balloon angioplasty and stent implantation were performed because the SMA lesion was considered a major treatable vascular contributor in a frail patient at high operative risk. Severe pain improved and oral intake advanced after revascularization, but portal-hypertensive disease, partial small-bowel obstruction, severe malnutrition, and SSc-related gastrointestinal involvement remained plausible concurrent contributors. Follow-up CTA in March 2024 showed post-stent changes without recurrent acute mesenteric ischemia, but long-term stent-patency and nutritional-recovery data were limited. This case does not establish a new causal mechanism; rather, it illustrates a structured approach to severe abdominal pain in SSc that distinguishes a treatable vascular lesion from SMAS-like anatomy and other nonvascular abdominal disease.

## Introduction

Systemic sclerosis (SSc) is a chronic autoimmune connective tissue disease characterized by vasculopathy, immune activation, and fibrosis. Gastrointestinal involvement is common and may include gastroesophageal reflux, dysmotility, intestinal pseudo-obstruction, bacterial overgrowth, malabsorption, and progressive nutritional decline (1–4). These manifestations are often sufficient to explain abdominal symptoms, which creates a diagnostic trap when a second and potentially reversible vascular process is present.

Chronic mesenteric ischemia is much less frequent than dysmotility in SSc, but it is clinically important because delayed diagnosis can lead to bowel infarction, severe malnutrition, and death. It usually reflects fixed stenosis or occlusion of major mesenteric vessels and classically presents with post-prandial pain, food avoidance, and weight loss (5, 6). Case reports suggest that patients with SSc can develop mesenteric ischemia from severe atherosclerotic disease or vasculopathy (7, 8), but the presentation may overlap with more familiar SSc-related gastrointestinal complications.

A separate entity is superior mesenteric artery syndrome (SMAS), in which loss of the mesenteric fat pad narrows the aortomesenteric angle and compresses the third part of the duodenum. SMAS-like anatomy may coexist with intrinsic SMA stenosis in severely malnourished patients, but the two processes have different diagnostic and therapeutic implications (9–11). The value of the present report is therefore not that chronic mesenteric ischemia or SMAS in SSc is unprecedented. Instead, the case provides an objective diagnostic sequence for a patient in whom high-grade intrinsic SMA stenosis, measured aortomesenteric narrowing, portal-hypertensive disease, partial obstructive features, and SSc gastrointestinal involvement all had to be weighed concurrently.

## Case description

A 72-year-old woman with Raynaud’s phenomenon for more than 30 years was admitted on 5 January 2024 for severe abdominal pain after approximately 2 months of progressive postprandial intake limitation and weight loss. Her SSc background was supported by mask-like facial sclerosis, radial perioral furrowing, sclerodactyly and distal acral skin thickening, telangiectasia (Figure 1), an antinuclear antibody titer of 1:320 with a centromere pattern, and strongly positive anticentromere antibody. The documented skin distribution was most consistent with limited cutaneous SSc; no trunk or proximal-limb skin thickening was recorded. A formal modified Rodnan skin score had not been prospectively documented and could not be reliably reconstructed retrospectively. Before this admission, she had not received regular glucocorticoid or immunosuppressive therapy. Previous treatment was mainly supportive and cardiopulmonary, including ambrisentan for pulmonary hypertension/right-heart-failure symptoms and diuretic therapy when clinically indicated. The key clinical timeline and decision points are summarized in Table 1.

**Figure 1:**
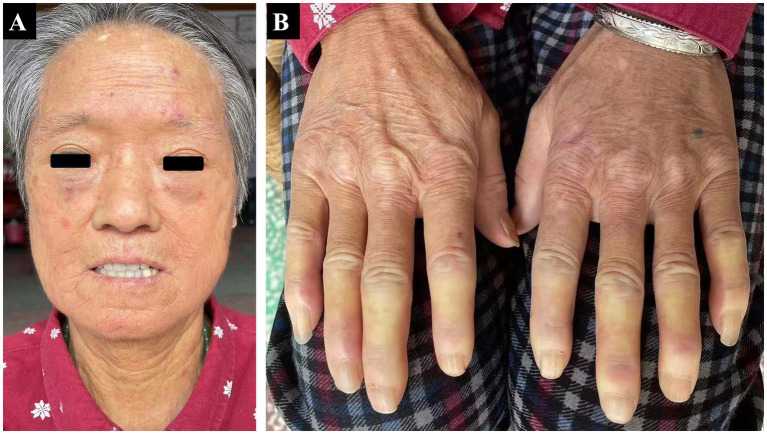
Cutaneous manifestations of systemic sclerosis. **(A)** Facial skin thickening with reduced wrinkles and radial perioral furrowing. **(B)** Digital sclerosis and Raynaud-related color change. The facial image has been de-identified by masking the eyes.

**Table 1:** Clinical timeline and key decision points.

Time or domain	Findings	Interpretation for the case report
>30 years before admission	Raynaud phenomenon, limited cutaneous SSc phenotype based on facial/acral involvement, sclerodactyly, telangiectasia, ANA centromere pattern, and anticentromere antibody positivity; no formal mRSS was prospectively recorded.	Established long-standing limited cutaneous SSc background; mRSS could not be reconstructed reliably from retrospective records.
2020–2022	Progressive dyspnea and recurrent right-heart-failure episodes; outside echocardiography reportedly estimated PASP at approximately 80 mmHg and later 66 mmHg; no right heart catheterization.	Pulmonary hypertension/right-heart-failure symptoms increased procedural risk; no right heart catheterization, so PAH classification remained unconfirmed.
Preceding 2 years	Body weight decreased from approximately 60 kg to 45 kg; BMI was about 17 kg/m2 at admission.	Major malnutrition increased risk for a narrowed aortomesenteric angle and complicated surgery.
5 January 2024 admission	Severe cramping abdominal pain after approximately two months; pain worsened after eating/upright position and was partly relieved in the left lateral position; profound anemia, hypoalbuminemia, elevated CRP, portal hypertension, ascites, and no interstitial lung disease on chest CT.	The vascular pain pattern and systemic risk profile justified urgent abdominal vascular imaging.
9 January 2024 abdominal CTA	Severe calcified stenosis at the SMA origin; moderate inferior mesenteric artery stenosis; mild celiac artery stenosis; renal artery stenoses; cirrhosis, splenomegaly, ascites, and splenic infarcts.	High-grade treatable SMA lesion plus SMAS-like anatomy; alternative abdominal contributors remained possible.
15–16 January 2024 multidisciplinary assessment and intervention	Multidisciplinary and vascular-surgical assessment on 15 January 2024 was followed by SMA angiography, balloon angioplasty, and stent placement on 16 January 2024; angiography showed >90% short-segment SMA origin stenosis and improved distal flow after stenting.	Endovascular treatment was favored over open surgery because of frailty and cardiopulmonary/hepatic risk.
18–19 March 2024 follow-up	Readmission for reassessment; abdominal CTA on 19 March showed post-stent changes at the SMA origin; severe ischemic pain did not recur, although intermittent mild discomfort and ascites persisted.	Short-term stent-related follow-up documented; long-term scheduled CTA patency and objective nutritional recovery data were unavailable.
25–28 March 2024 additional findings	PET-CT suggested partial small-bowel obstruction and showed a bladder lesion; transurethral tumor resection on 28 March revealed low-grade papillary urothelial carcinoma.	Persistent symptoms were not attributed automatically to recurrent ischemia; competing abdominal diagnoses were evaluated.
Following months and July 2025	Family reported gradual reduction of abdominal pain over the following months. The patient later died at another hospital in July 2025 during treatment for cardiopulmonary failure.	Late outcome could not be causally linked to the SMA lesion or stent; underscores limited long-term outcome data.

This table summarizes the major clinical milestones, diagnostic findings, therapeutic decisions, and follow-up outcomes. The interpretation column highlights how each time point contributed to the diagnosis of chronic mesenteric ischemia, recognition of coexisting SMAS-like anatomy, and selection of endovascular treatment.

ANA, antinuclear antibody; BMI, body mass index; CRP, C-reactive protein; CT, computed tomography; CTA, computed tomography angiography; GI, gastrointestinal; PASP, pulmonary artery systolic pressure; PAH, pulmonary arterial hypertension; PET-CT, positron emission tomography-computed tomography; SMA, superior mesenteric artery; SMAS, superior mesenteric artery syndrome; SSc, systemic sclerosis; SSc-PAH, systemic sclerosis-associated pulmonary arterial hypertension.

From 2020 onward, she developed progressive exertional dyspnea and episodes of right-heart failure. Outside echocardiography reportedly estimated pulmonary artery systolic pressure at approximately 80 mmHg in 2021 and 66 mmHg in 2022. Right heart catheterization had not been performed. She was also found to have cirrhosis, portal hypertension, splenomegaly, cytopenias, and recurrent ascites. Viral hepatitis tests were negative in the available records. Primary biliary cholangitis was clinically considered because of the anticentromere-positive SSc phenotype, but antimitochondrial antibody testing and liver histology were not available for confirmation (12, 13). During the two years before the vascular presentation, body weight decreased from approximately 60 kg to 45 kg.

On admission in January 2024, she was cachectic, with a body mass index of approximately 17 kg/m2. Physical examination showed mask-like facial sclerosis, radial perioral furrowing, sclerodactyly, telangiectasia, abdominal distension, shifting dullness, and mild lower-limb edema. Key laboratory abnormalities are summarized in Table 2 and included severe anemia, hypoalbuminemia, elevated inflammatory markers, iron and folate deficiency, anticentromere antibody positivity, and elevated NT-proBNP. These findings supported severe systemic frailty and active abdominal stress, but they did not by themselves identify a single cause of pain.

**Table 2:** Concise laboratory and clinical data supporting the diagnostic assessment.

Domain	Representative findings	Interpretation in diagnostic reasoning
Hematologic and nutritional status	Hemoglobin approximately 50–57 g/L during early January 2024; albumin approximately 26.8–27.7 g/L; iron and folate deficiency; BMI approximately 17 kg/m2.	Severe frailty and malnutrition increased procedural risk and could contribute to SMAS-like anatomy and nonspecific abdominal symptoms.
Inflammation and infection screen	CRP markedly elevated during the index admission; procalcitonin low to mildly increased; no single infectious focus established.	Supported active abdominal/systemic stress but was not specific for chronic mesenteric ischemia.
SSc serology and phenotype	ANA 1:320 with centromere pattern; anticentromere antibody strongly positive; facial/acral skin involvement without documented trunk or proximal-limb thickening.	Consistent with limited cutaneous SSc; formal mRSS was unavailable.
Cardiopulmonary risk	Elevated NT-proBNP and echocardiographic pulmonary hypertension; right heart catheterization not performed.	Important for risk stratification but insufficient for definitive PAH classification.
Hepatic and portal-hypertensive context	Cirrhotic liver morphology, splenomegaly, ascites, hypoalbuminemia, and cytopenias.	Plausible competing contributors to pain, frailty, and procedural risk; liver etiology remained uncertain.
Follow-up laboratory context	Persistent anemia, hypoalbuminemia, and frailty during March–April 2024 follow-up.	Short-term symptomatic improvement did not establish complete nutritional recovery or long-term efficacy.
Hematologic and nutritional status	Hemoglobin approximately 50–57 g/L during early January 2024; albumin approximately 26.8–27.7 g/L; iron and folate deficiency; BMI approximately 17 kg/m2.	Severe frailty and malnutrition increased procedural risk and could contribute to SMAS-like anatomy and nonspecific abdominal symptoms.

ANA, antinuclear antibody; BMI, body mass index; CRP, C-reactive protein; mRSS, modified Rodnan skin score; PAH, pulmonary arterial hypertension; SSc, systemic sclerosis.

## Diagnostic assessment, therapeutic intervention, follow-up and outcomes

Because the severity, recurrence, food-related worsening, positional features, and nutritional decline were disproportionate to ordinary SSc dysmotility, abdominal CTA was performed on 9 January 2024. CTA showed severe short-segment calcified stenosis at the SMA origin, estimated at greater than 90%, with additional moderate inferior mesenteric artery stenosis, renal arterial stenoses, a cirrhotic-appearing liver, splenomegaly, ascites, and a splenic infarct. Multiplanar CTA review also showed reduced aortomesenteric measurements, with an angle of 17 degrees and distance of 4.7 mm (Figure 2), compatible with a SMAS-like anatomic context. The diagnostic reasoning therefore treated chronic mesenteric ischemia as an important and actionable vascular contributor rather than as the only possible explanation. Portal hypertension, partial small-bowel obstruction, severe malnutrition, and SSc-related gastrointestinal involvement were retained as competing or concurrent contributors. A practical framework for this parallel diagnostic assessment is shown in Figure 3.

**Figure 2:**
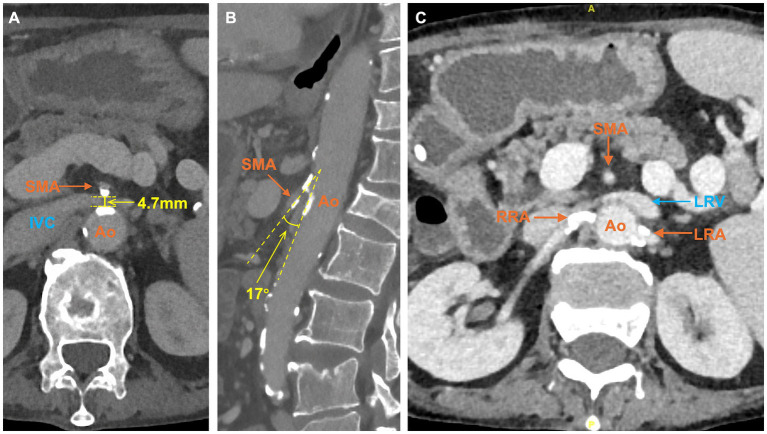
Abdominal CT angiography. **(A)** Axial CTA shows a reduced aortomesenteric distance of 4.7 mm. **(B)** Sagittal reconstruction shows a reduced aortomesenteric angle of 17 degrees. **(C)** Axial CTA shows the SMA origin and adjacent abdominal vessels. The measured SMAS-like anatomy coexisted with intrinsic high-grade SMA origin stenosis and should not be interpreted as the sole mechanism of ischemic pain. Ao, abdominal aorta; SMA, superior mesenteric artery; IVC, inferior vena cava; RRA, right renal artery; LRA, left renal artery; LRV, left renal vein.

**Figure 3:**
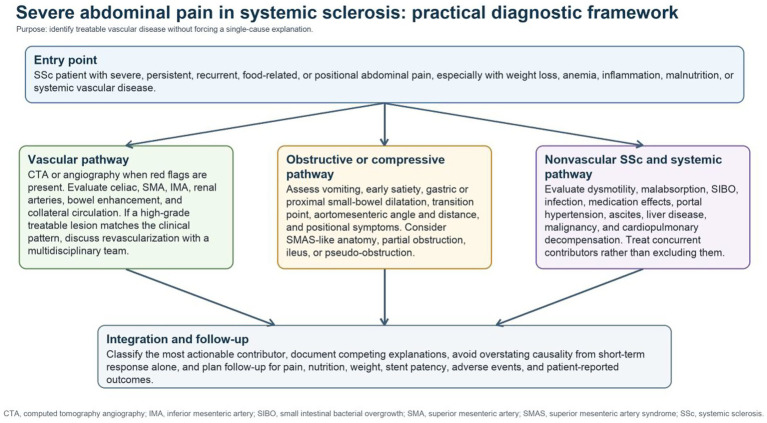
Practical diagnostic framework for severe abdominal pain in systemic sclerosis. The framework emphasizes parallel assessment of treatable vascular disease, obstructive or SMAS-like anatomy, and nonvascular SSc or systemic contributors, followed by integrated multidisciplinary management and cautious follow-up interpretation.

A multidisciplinary team including rheumatology, vascular surgery, gastroenterology, cardiology, radiology, nutrition, and nursing services reviewed the case. Endovascular therapy was chosen because ongoing ischemic pain and malnutrition created immediate risk, whereas open surgery was considered high risk in a frail patient with pulmonary hypertension, portal hypertension, anemia, hypoalbuminemia, and severe systemic illness. After multidisciplinary and vascular-surgical assessment on 15 January 2024, the patient underwent abdominal aortography, SMA angiography, balloon angioplasty, and SMA stent placement on 16 January 2024. Intraoperative angiography showed severe short-segment stenosis of more than 90% at the SMA origin with calcified plaque; after stenting, the residual stenosis was mild and distal mesenteric blood flow improved.

The procedure was uncomplicated. After the intervention, severe abdominal pain improved substantially, the patient tolerated small amounts of semi-liquid and soft food, and overt postprandial severe pain did not recur during the index hospitalization. This temporal association supported the clinical relevance of the SMA lesion, but it was not interpreted as definitive proof that chronic mesenteric ischemia was the sole cause of abdominal pain. She received nutritional support, anti-infective therapy when clinically indicated, transfusion support, and cardiopulmonary management. Antiplatelet therapy was initiated after stenting, but clopidogrel was discontinued because of bleeding risk and anemia; aspirin was continued. Ambrisentan was used during the broader SSc/cardiopulmonary management plan (14), and low-dose glucocorticoid therapy was used cautiously because corticosteroid exposure in SSc requires attention to renal-crisis risk (15, 16).

During early follow-up, severe ischemic-type abdominal pain did not recur, although intermittent mild spasm-like abdominal discomfort persisted. She was readmitted on 18 March 2024, and abdominal CTA on 19 March 2024 showed post-stent changes at the SMA origin without evidence of recurrent acute mesenteric ischemia. Subsequent PET-CT on 25 March 2024 showed small-bowel wall thickening with proximal dilatation and fluid levels, suggesting that partial obstruction or severe dysmotility could still contribute to symptoms. Ascites drainage and supportive management were performed. At outpatient/telephone follow-up, the family reported that abdominal pain gradually decreased during the following months and that no further SMA intervention was performed, but scheduled long-term CTA surveillance, objective weight-gain documentation, and formal nutritional outcome measurements were unavailable. The family later reported that the patient died in July 2025 at another hospital during treatment for cardiopulmonary failure; the relationship of this late outcome to the SMA lesion or stent could not be determined.

## Discussion

This case is clinically useful because it shows how severe abdominal pain in SSc can require simultaneous evaluation of vascular, obstructive, portal-hypertensive, nutritional, and motility-related mechanisms. The case should not be read as proving that high-grade SMA stenosis was the exclusive cause of pain. Rather, the combination of a vascular pain phenotype, CTA-confirmed high-grade SMA origin stenosis, high operative risk, and short-term improvement after revascularization supported classifying the lesion as the dominant treatable contributor within a multifactorial abdominal illness (3–6).

The literature supports separating two vascular concepts. Reports by Barasa and Bader and by Hong et al. describe SSc patients with mesenteric ischemia related to mesenteric arterial disease (7, 8), whereas reports by Chua et al., Gowda et al., and Saeed et al. emphasize SMAS or proximal intestinal obstruction in SSc or severe weight loss (9–11). The focused comparison with published reports is summarized in Table 3. The incremental contribution of the present case is narrower and more objective than claiming novelty by rarity: it documents measured aortomesenteric narrowing coexisting with fixed high-grade SMA origin stenosis and uses this overlap to show why CTA-based differential reasoning may be necessary before attributing pain to SSc dysmotility alone.

**Table 3:** Published SSc reports and the focused clinical contribution of the current case.

Report	Main vascular or obstructive finding	Key comparison with the current case
Barasa and Bader, 2023 (7)	Chronic mesenteric ischemia in limited SSc with mesenteric arterial stenosis.	Supports that fixed mesenteric arterial disease can occur in SSc and mimic GI dysmotility.
Hong et al., 2022 (8)	Mesenteric ischemia described as a rare complication of scleroderma.	Shows the severity of mesenteric ischemia in SSc, but details differ from this endovascularly treated case.
Chua et al., 2020 (9)	SMAS in SSc with duodenal compression after severe weight loss.	Represents the compression/obstruction category, not intrinsic SMA origin stenosis.
Gowda et al., 2024 (10)	Two diffuse SSc cases with SMAS and marked narrowing of the aortomesenteric angle/distance.	Supports severe weight loss as a mechanism for SMAS-like obstruction in SSc.
Saeed et al., 2020, conference abstract (11)	Small intestinal obstruction from SMAS in SSc.	Again, emphasizes obstruction from compression rather than calcified arterial stenosis.
Current case	High-grade calcified SMA origin stenosis confirmed by CTA and angiography, with measured aortomesenteric narrowing (17 degrees and 4.7 mm) and concurrent portal-hypertensive, nutritional, obstructive, and SSc gastrointestinal considerations.	Documents measured aortomesenteric narrowing coexisting with fixed high-grade SMA origin stenosis and illustrates structured differential reasoning in a multifactorial SSc abdomen. The case supports considering CTA in selected high-risk patients but does not establish frequency, exclusivity, or causality.

This table compares previously published systemic sclerosis cases involving mesenteric ischemia or SMAS with the current case. The comparison is intended to define a focused educational contribution, not to claim that the association is unprecedented or to establish causality.

CTA, computed tomography angiography; GI, gastrointestinal; SMA, superior mesenteric artery; SMAS, superior mesenteric artery syndrome; SSc, systemic sclerosis.

This distinction changes the diagnostic pathway. SMAS is usually suspected when vomiting, early satiety, gastric or proximal duodenal dilatation, and postural improvement dominate the presentation (9–11). Chronic mesenteric ischemia is more likely when severe postprandial or food-limiting pain, weight loss, and vascular calcification or stenosis are present (5, 6). In SSc, these patterns may overlap with pseudo-obstruction, small intestinal bacterial overgrowth, malabsorption, portal hypertension, ascites, infection, or medication-related symptoms (3, 4, 17, 18). Figure 3 summarizes a practical diagnostic framework that keeps these alternatives active while identifying treatable vascular disease.

SSc may still be relevant to the vascular phenotype. Chronic endothelial injury, vascular remodeling, inflammation, and accelerated atherosclerosis have been reported in SSc (1, 2, 15, 21). In an older patient with calcified plaques, hypertension, diabetes history, and coronary and renal artery calcification, however, it is not possible to prove that SSc caused the SMA stenosis. The most balanced interpretation is that long-standing SSc, systemic illness, malnutrition, and conventional atherosclerotic disease coexisted and together increased the likelihood that mesenteric arterial stenosis became symptomatic.

Pulmonary hypertension and liver disease complicated diagnosis and risk assessment but were not the main message of the report. Right heart catheterization was not performed, so pulmonary arterial hypertension cannot be definitively classified according to contemporary pulmonary hypertension standards (19). Likewise, the etiology of liver disease remained uncertain; primary biliary cholangitis, congestive hepatopathy, portopulmonary disease, and pulmonary-hypertension-related liver abnormalities were considered, but confirmatory serology or histology was unavailable (12, 13, 17, 18, 20). For this reason, both topics are presented as important comorbidities and competing contributors to abdominal symptoms rather than as resolved mechanistic explanations.

The therapeutic narrative therefore centers on individualized risk management. Endovascular treatment addressed a high-grade and potentially reversible SMA lesion while avoiding the morbidity of open surgery in a malnourished patient with cardiopulmonary and hepatic comorbidity. At the same time, persistent ascites, later imaging features suggesting partial obstruction or severe dysmotility, and incomplete nutritional recovery data argue against overstating the long-term efficacy of the vascular intervention.

The main limitations are the absence of right heart catheterization, the lack of definitive histologic or serologic clarification of the liver disease, lack of formal baseline mRSS documentation, incomplete long-term stent-patency surveillance, limited objective nutritional and weight-recovery data, and the inability of a single case to establish causality. The short-term response after revascularization is clinically informative but cannot exclude concurrent portal-hypertensive, obstructive, malnutrition-related, or SSc gastrointestinal contributors.

## Patient perspective

A formal written patient perspective was not recorded. During clinical follow-up, the patient and family emphasized that relief of severe food-related abdominal pain and the ability to resume oral intake were the most meaningful early outcomes of treatment. Written informed consent was obtained for publication of the de-identified clinical information and images.

## Conclusion

In patients with SSc, severe or persistent abdominal pain with marked weight loss should prompt early consideration of abdominal vascular imaging. High-grade SMA origin stenosis can be a treatable cause of chronic mesenteric ischemia even when SSc-related gastrointestinal dysmotility, portal-hypertensive disease, and reduced aortomesenteric angle and distance coexist. Distinguishing intrinsic SMA stenosis from SMAS-like compression is central to safe clinical interpretation and treatment planning.

## Data Availability

The original contributions presented in the study are included in the article/Supplementary material, further inquiries can be directed to the corresponding author.
