# Supplementary material for: Case Report: Vascular abdominal pain in systemic sclerosis—chronic mesenteric ischemia from high-grade superior mesenteric artery stenosis with coexisting aortomesenteric narrowing
Source: Front Med (Lausanne). 2026 Jul 24;13:1882228. doi: 10.3389/fmed.2026.1882228 (PMC13447209; doi:10.3389/fmed.2026.1882228)
Supplement: Supplementary file 1 [file Table_1.DOCX]

**Supplementary Material: CARE Checklist**

Manuscript: Severe abdominal pain in limited systemic sclerosis: high-grade superior mesenteric artery stenosis with coexisting aortomesenteric narrowing

| **CARE item** | **Location in manuscript** | **Comment** |
| --- | --- | --- |
| Title | Title page | Identifies the article as a case report and states the core clinical problem. |
| Keywords | Keywords | Includes systemic sclerosis, chronic mesenteric ischemia, SMA stenosis, SMAS, and abdominal pain. |
| Abstract | Abstract | Summarizes presentation, diagnostic assessment, intervention, outcome, and learning point. |
| Introduction | Introduction | Explains clinical context and focused educational contribution. |
| Patient information | Case description | Provides age, sex, SSc phenotype, major symptoms, comorbidities, and prior treatment context. |
| Clinical findings | Case description and Table 1 | Summarizes skin findings, abdominal findings, cardiopulmonary risk, and nutritional status. |
| Timeline | Table 1 | Presents key dates and decision points from admission through follow-up. |
| Diagnostic assessment | Diagnostic assessment, Table 2, Figures 2-3 | Describes CTA findings, laboratory context, differential diagnosis, diagnostic reasoning, and limitations. |
| Therapeutic intervention | Therapeutic intervention | Describes endovascular intervention, supportive care, medications, and antiplatelet adjustment. |
| Follow-up and outcomes | Follow-up and outcomes; Table 1 | Reports short-term clinical and imaging follow-up and limitations of long-term outcome data. |
| Discussion | Discussion | Discusses strengths, limitations, competing explanations, literature context, and practical diagnostic approach. |
| Patient perspective | Patient perspective | Reports patient/family perspective available from clinical follow-up. |
| Informed consent | Ethics statement | States written informed consent and ethics review. |
